# Dexmedetomidine to Help Nerve Regeneration in a Rat Sciatic Nerve Injury Model

**DOI:** 10.1155/2017/9045608

**Published:** 2017-12-13

**Authors:** Wook Jeong, Hsichiang Kung, Chia Chi Cheng, Changwoo Lim, Min Jung Jung, Jaeho Lee, Doo Sik Kim, Yusom Shin

**Affiliations:** ^1^Department of Anesthesiology and Pain Medicine, Suryeoan Clinic, Busan, Republic of Korea; ^2^Department of Anesthesiology and Pain Medicine, Kosin University College of Medicine, Busan, Republic of Korea; ^3^Department of Pathology, Kosin University College of Medicine, Busan, Republic of Korea; ^4^Department of Anesthesiology and Pain Medicine, Ulsan University College of Medicine, Ulsan, Republic of Korea

## Abstract

**Background:**

Several studies have shown that dexmedetomidine (DXM), a selective *α*2-adrenoceptor agonist, also has neuroprotective effects. However, its effect on impaired peripheral nerve regeneration has not been studied.

**Materials and Methods:**

Forty-five Sprague-Dawley rats were randomly assigned to three groups: group 1 (control SHAM), group 2 (sciatic nerve injury + normal saline), and group 3 (sciatic nerve injury + DXM). The rats of group 3 were subdivided into the following three groups: DXM 0.5, 6, and 20 *μ*g·kg^−1^ (groups 3A, 3B, and 3C, resp.). The sciatic nerve injury was assessed for nerve regeneration at 2 and 6 weeks.

**Results:**

There were no differences between groups 2 and 3 in their sciatic functional index (SFI) values or histological findings at 2 weeks postinjury. However, SFI differences were statistically significant at 6 weeks postinjury in group 3. The gross findings with H&E staining showed that the number of axons was higher in group 3 than in group 2. There was no histological difference according to the DXM concentration.

**Conclusion:**

The coincidental functional and histological assessment results of this study suggest that DXM for 6 weeks positively affects damaged peripheral nerves.

## 1. Introduction

Peripheral nerve injury requires a long recovery period, and recovery, once attained, usually is incomplete [1]. Peripheral nerve damage can, by manifesting as serious disability, negatively impact upon patients' daily life as well as quality of life (QoL). Indeed, patients might lose as much as 21% of their accustomed daily activities, and such activity loss is strongly associated with depression. And certainly, the higher the pain, disability, and depression levels, the worse the QoL [2, 3]. Crush injuries are more likely to be accompanied by peripheral nerve injuries, which are sustained most commonly by young people aged between 21 and 30 [4]. Management for recovery of peripheral nerve injury is important in terms of not only QoL but also medical (and therefore social) costs.

Dexmedetomidine (DXM), a selective *α*2-adrenergic receptor agonist that has an eightfold affinity to the receptor relative to clonidine, has anxiolytic, sedative, and analgesic effects. Widely used in anesthesia and intensive care medicine, DXM's characteristic sedative effect appears without respiratory suppression. Its most common side effects are bradycardia and hypotension. Animal models and human studies show evidence of potential neuroprotective effects on the brain [5]. In the developing rat brain, for example, DXM contributes to neuroprotection via effectively reducing ketamine-induced or propofol-induced neuroapoptosis [6, 7].

However, whereas its neuroprotective effects have been well documented, there is as yet no data on any peripheral nerve neuroprotective effects. If it can be shown that DXM also has a neuroprotective effect on the peripheral nerve, it will have possible uses for the treatment of damaged peripheral nerves. Based on evidence that DXM does not promote perineural inflammation or local nerve damage when administered perineurally to uninjured sciatic nerves of rats [8], the present study aimed to determine the effects of different doses of perineural DXM on nerve regeneration in a rat model of sciatic nerve crush injury.

## 2. Materials and Methods

### 2.1. Study Animals

This study was approved by the Ethical Committee of Kosin University College of Medicine, Busan, Korea. Forty-five (45) 12-week-old Sprague-Dawley (SD) rats weighing approximately 300 to 400 g were used in the experiments. The rats were assigned two for each cage and maintained under conditions of controlled temperature (20 ± 2°C) and humidity (40–60%) on a 12 hour light/dark cycle with sufficient water and feed. The rats were randomly divided into three groups: a surgical incision without nerve injury (group 1; *n*=9), a nerve injury with normal saline injection (group 2; *n*=9), and a nerve injury with DXM injection (group 3). The group 3 rats were divided into three subgroups: DXM 0.5, 6, and 20 *μ*g·kg^−1^ (group 3A, 3B, and 3C; *n*=9, resp.).

### 2.2. Surgical Procedure

All of the rats were anesthetized with a single intraperitoneal injection of tiletamine-zolazepam (Zoletil 50, Virbac, France) at a dose of 60 mg/kg body weight mixed with xylazine (Rompun, Bayer, Korea) at a dose of 5 mg/kg body weight; additional doses of this mixture were administered as needed. After anesthesia, the left-side buttock and thigh area was shaved and sterilized with povidone-iodine solution. For exposure of the sciatic nerve, an incision was made on the line between the knee joint and the ischial tuberosity, and the biceps femoris muscle was incised. If the left sciatic nerve was detected, it was crushed into about a 4 mm length using a curved Kelly hemostatic clamp for 30 seconds with a clamping force of approximately 40 N before its bifurcation. Then, normal saline or dexmedetomidine was injected perineurally according to the group to which each rat belonged. Finally, the skin was sutured with 4-0 stitches.

### 2.3. Dexmedetomidine Administration

**Figure 1:**
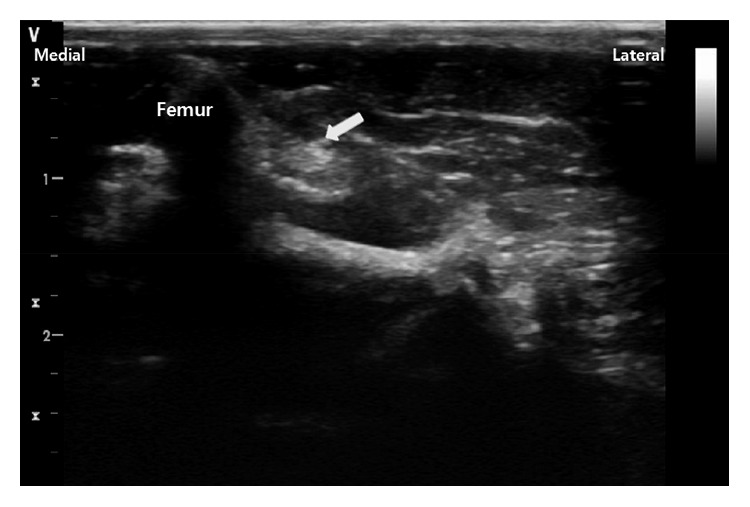
Ultrasound image of sciatic nerve (arrow).

**Figure 2:**
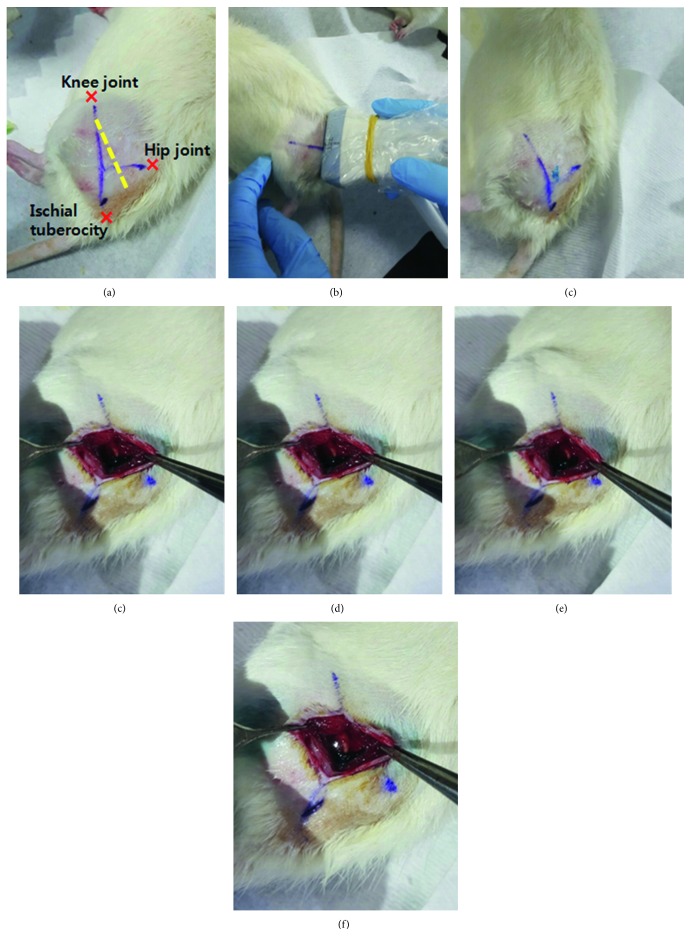
Perineural dexmedetomidine injection under ultrasound guidance. (a) Expected path of sciatic nerve marked with yellow dotted line. The sciatic nerve runs between the hip joint and the ischial tuberosity toward the knee joint. (b) Sciatic nerve detection by placement of probe obliquely or vertically on expected course. An image of the nerve as detected via ultrasound is shown in Figure 1. (c) Blue dye injection using same volume of DXM (0.08 ml) in experiment. The entry point is indicated by the blue spot. (d–f) A series of photographs of post-blue-dye-injection. After incising the skin between hip joint and ischial tuberosity and dissecting fascia and muscle, blue dye is visible around the sciatic nerve.

The 27 rats of group 3, once anesthetized, underwent perineural DXM 0.5, 6, and 20 *μ*g·kg^−1^ injection (groups 3A, 3B, and 3C, resp.). During this study, scheduled for treatment at two and six weeks, DXM administration was performed three times per week. Injection around the exposed left sciatic nerve with crush injury was begun immediately upon sciatic nerve crush injury. On postoperative day 2, the same process was repeated, with the additional purpose of confirming nerve injury. From the third injection, DXM was injected under ultrasound guidance without skin incision. The target, the left sciatic nerve, was observed and contacted with a gel-applied linear probe in its right lateral position (Figure 1). The expected injection site was wiped with an alcohol-applied pad. A 26 G needle with a 1 ml syringe was placed near the sciatic nerve using the out-of-plane approach, and 0.08 ml of DXM 0.5, 6, and 20 *μ*g·kg^−1^ was injected into the rats of each group (Figure 2). In group 2, 0.08 ml normal saline was administered in the same manner.

### 2.4. Functional Analysis

**Figure 3:**
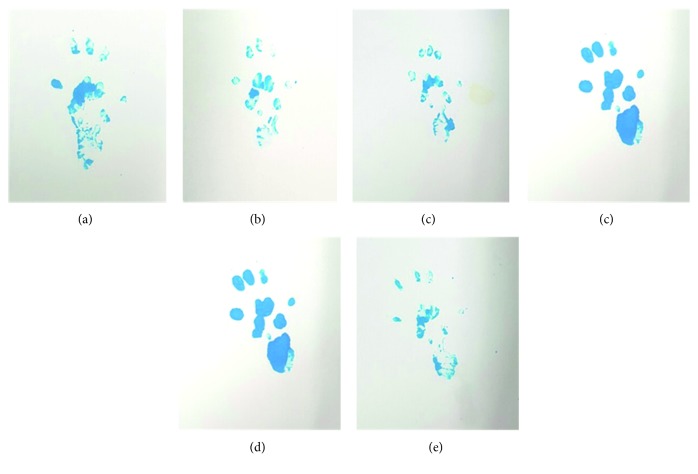
Rat walking track (experimental). (a) Group 1: surgical incision without nerve injury. (b) Group 2: nerve injury with normal saline injection. (c–e) Group 3: nerve injury with 0.5, 6, and 20 *μ*g·kg^−1^ DXM injection (groups 3A, 3B, and 3C, resp.).

Functional recovery of the nerve was evaluated by the sciatic functional index (SFI), which was obtained from walking-track analyses at the 2- and 6-week treatments, respectively. All of the rats entered into a dark single-channel 100 × 10 cm closed box with blue ink on their feet. Their footprints were obtained on white paper laid on the floor of the box (Figure 3). The footprints provided the lengths of three parameters: (i) heel to third toe (print length, PL), (ii) first toe to fifth toe (toe spread, TS), and (iii) second toe to fourth toe (intermediate toe spread, ITS). The SFI was calculated by the following equations: SFI = (−38.3 × PLF) + (109.5 × TSF) + (13.3 × ITSF) − 8.8, print length factor (PLF) = (EPL – NPL)/NPL, toe spread factor (TSF) = (ETS − NTS)/NTS, and intermediate toe spread factor (ITSF) = (EITS − NITS)/NITS. Herein, *E* is the tested foot, and *N* is the normal foot. The higher the SFI value, the higher the degree of functional recovery. Generally, an SFI value close to −100 indicates complete impairment, while a value close to 0 implies normal function.

### 2.5. Histological Analysis

The sciatic nerves of two rats from each group were obtained under anesthesia before sacrifice. The specimens were fixed in neutral-buffered formalin 10% solution and sliced into about 5 *μ*m thicknesses as vertically as possible. All of the slides were stained with haematoxylin and eosin (H&E) and observed under light microscopy. The total axon count was calculated by only one pathologist.

### 2.6. Statistical Analysis

The experimental data are presented as medians. A statistical analysis was performed using MedCalc (ver. 17.5.5). Statistical significance was assessed by the Mann-Whitney and Friedman tests. *P* values less than 0.05 were considered statistically significant.

## 3. Results

### 3.1. Functional Recovery after Sciatic Nerve Injury

**Figure 4:**
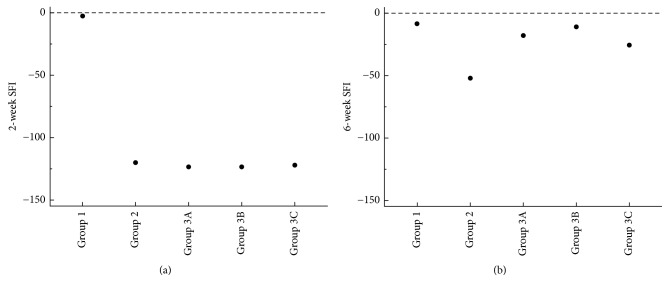
Median SFI values (dots) at 2 and 6 weeks for all groups. At 2 weeks postoperatively, the SFI value of group 1 is close to zero, indicating an uninjured sciatic nerve (*P*=0.02, statistical comparison between groups 1 and 2 via the Mann-Whitney test). In groups 2 and 3 (A, B, and C), all of the SFI values exceed −100, indicating total impairment. At 6 weeks postoperatively, all of the median SFI values for group 3 are higher than those for group 2 (*P*=0.02, statistical comparison among all groups via the Friedman test).

The SFI values were measured at 2 and 6 weeks postoperatively (Figure 4). At 2 weeks, the medians of the SFI values were similar among all of the groups except group 1, which indicated total impairment of the sciatic nerve. At 6 weeks, the medians of the SFI values were improved among the entire group 3 (the medians of groups 3A, 3B, and 3C; −17.856, −10.676, and −25.564, resp.); moreover, a significant difference in the SFI values between groups 1 and 2 was statistically confirmed (*P*=0.02). It was determined that group 3 was statistically different from all of the other groups in the 2-week and 6-week treatment groups (*P*=0.03).

### 3.2. Histological Assessment

**Figure 5:**
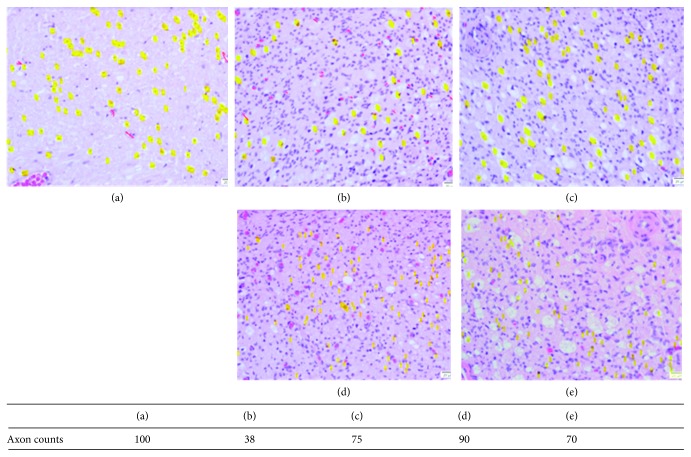
H&E staining of sciatic nerves in different groups at 6 weeks post nerve injury. The number of axons (yellow marks) was counted in a representative high-power field of the transverse section (×1000). (a) Group 1: surgical incision without nerve injury. (b) Group 2: nerve injury with normal saline injection. (c–e) Group 3: nerve injury with 0.5, 6, and 20 *μ*g·kg^−1^ DXM injection (groups 3A, 3B, and 3C, resp.).

The numbers of axons were quantified from images obtained under light microscopy. There were more axons in the injured sciatic nerves treated with DXM (groups 3A, 3B, and 3C) than in those of DXM-untreated group 2 (Figure 5). In group 3 (Figures 5–5), closely arranged and thinly myelinated regenerating fibers and normal myelinated fibers were evident. In group 2 (Figure 5), there were fewer axons with globular change in myelin.

## 4. Discussion

Among the many peripheral nerve injuries, incomplete injury is the most common [9]. Even when the nerve has sustained a crush injury by acute traumatic stress, it is often not completely disconnected [10]. Seddon classified crush nerve injury into three categories according to local myelin damage and neuropraxia maintaining the continuity of the axon [9]. Reinnervation into the denervated end-organ occurs in one of two ways: (1) collateral branching of the intact axons or (2) regeneration of the injured axon. When 20–30% of axons are damaged, recovery mainly entails the first process. By this mechanism, additional axonal branches begin to sprout on the 4th day postinjury and continue to do so for 3–6 months until recovery. When more than 90% of axons are damaged, recovery mainly entails the second process. Traumatic axonal damage causes loss of cell membrane integrity, breakdown of the axonal cytoskeleton, and formation of a microenvironment favoring axonal regrowth through Wallerian degeneration, all within one week of injury. It should not be overlooked, however, that reinnervation is not equivalent to complete functional recovery [11, 12].

The proximal mechanisms of DXM-effected neuroprotection include antiinflammation, inhibition of sympathetic activation via *α*2-adrenergic receptor activation, and activation of certain protective signal pathways such as Erk [13]. DXM also might contribute to neuroprotection by inhibiting calcium entry, scavenging glutamate, decreasing NMDA receptor activation, and increasing expression of growth factors [6, 13, 14]. Brimonidine, a selective *α*2-adrenergic receptor agonist, has a neuroprotective effect via the upregulation of neurotrophic growth factor and the neurotrophin signaling of Erk1/2 activation [15]. Perineural clonidine, a *α*2-adrenoceptor agonist, has been shown to relieve neuritis-induced pain and proinflammatory cytokines by transforming cytokine gene expression in macrophages and lymphocytes in peripheral nerve injury rats [16]. Although xylazine, another *α*2-adrenergic receptor agonist used herein as an agent for anesthesia, has been reported to promote axonal regeneration in damaged optic nerves [17], bias against it can be considered to have been excluded, as all of the rats were anesthetized. The rats in the group using xylazine alone for anesthesia did not show better results than those in other groups using DXM, either. In this experiment, which was initiated on the assumption that DXM with its neuroprotective mechanism might also be operative in peripheral nerve injury, DXM exhibited significant positive effects on post-crush-injury peripheral nerve regeneration.

It has been proved that perineural DXM used together with local anesthetics increases the duration of sensory and motor block in animal studies; in fact, DXM sustains spinal or brachial plexus block longer as a potential adjuvant to local anesthetics [18, 19]. Moreover, its addition to ropivacaine administered to volunteers perineurally prolonged peripheral nerve block by 60%, with no adverse effects [20]. DXM's three concentrations administered in the current study (0.5, 6, and 20 *μ*g·kg^−1^) had been selected according to the results of an animal study dose-dependently evaluating it as an adjuvant to local anesthetics, because it was necessary to choose comparable concentrations that would not cause any perineural inflammation to damaged nerves [21]. Regarding the duration-dependent side effects of DXM, it has been proven to be safe to use for a long time with high dosage [22, 23]. In this experiment, DXM was almost used every other day without continuous infusion. Considering its mean elimination half-life (2–2.5 hours), six-week usage of DXM is completely unrelated to side effects of long-term administration. Therefore, the side effects of DXM were not taken into consideration. In fact, at weeks 2 and 6, there were no rats with symptoms of DXM adverse events over time.

The nerve regeneration for each DXM concentration was evaluated according to its functional and histological aspects. Although von Frey, hot-late, and cold-plate tests are a good way to research for neuropathic pain, the SFI, a reliable tool for evaluation of post-sciatic-nerve-injury motor function in animal experiments, is commonly used for recent studies about sciatic nerve functional assessment [2, 24–26]. The SFI values for the DXM-treated group were similar to those of the DXM-untreated group at 2 weeks postinjury, indicating a quite unrecovered nerve. However, at 6 weeks postinjury, the DXM-treated groups showed better SFI values than those of the DXM-untreated group. Histologically, the number of axons on each slide was calculated using H&E staining, and higher axon counts were found on all of the slides of the DXM-treated groups than on those of the DXM-untreated group, with no confirmation of statistical significance. Due to the limitations of H&E staining, there were no gross-finding differences associated with the three concentrations of DXM. Authors could not choose better specimen analysis method than H&E staining due to our experimental conditions. It is certain that more information might have been obtained if a molecular biology method was used. The better SFI values in the DXM-treated groups implied better nerve regeneration, as was consistent with the histological findings.

It seems, therefore, that DXM has a positive effect on peripheral nerve regeneration. Determination or explication of the specific mechanisms involved was beyond the scope of the present experiment. Nonetheless, it is reasonable to speculate that the same DXM mechanisms as are involved in neuroprotection can be applied to peripheral nerve regeneration. Despite the limitations of this study, it is significant that the potential of DXM as a tool for management of damaged peripheral nerve was confirmed by its positive peripheral nerve regeneration effect.

## 5. Conclusion

Administration of DXM around the injured peripheral nerve might facilitate peripheral nerve regeneration. In this study, there were no significant differences among the DXM concentrations, and so it is advantageous, in terms of possible side effects, that DXM administration can be started at the lowest available dose. Also, prolonged administration of it is recommended, as DXM showed no signs of any contribution to nerve recovery at 2 weeks treatment. It will be necessary to confirm the present results in a more detailed injection concentration and duration setting.
